# Fear of COVID-19, Mindfulness, Humor, and Hopelessness: A Multiple Mediation Analysis

**DOI:** 10.1007/s11469-020-00419-5

**Published:** 2020-11-19

**Authors:** Mehmet Saricali, Seydi Ahmet Satici, Begum Satici, Emine Gocet-Tekin, Mark D. Griffiths

**Affiliations:** 1 Department of Psychology, Nevşehir Hacı Bektaş Veli University, Nevşehir, Turkey; 2 Department of Psychological Counselling, Artvin Coruh University, Artvin, Turkey; 3 Department of Foreign Languages, Sakarya University, Sakarya, Turkey; 4 International Gaming Research Unit, Psychology Department, Nottingham Trent University, 50 Shakespeare Street, Nottingham, NG1 4FQ UK

**Keywords:** COVID-19, Hopelessness, Mindfulness, Humor, Fear of COVID-19, Turkey

## Abstract

Hopelessness is an important vulnerability factor for depressive symptomology and suicidal ideations. It may also play an important role in the fear of coronavirus 2019 (COVID-19). Therefore, the present study tested the mediating role of mindful awareness and humor (both identified as coping strategies for dealing with stressful situations) in the relationship between fear of COVID-19 and hopelessness. Participants comprised 786 Turkish individuals (562 females and 224 males; aged between 18 and 67 years) from 71 of 81 cities in Turkey. An online convenience sampling method was used to recruit participants. Participants completed surveys including the Fear of COVID-19 Scale, Beck Hopelessness Scale, Mindful Attention Awareness Scale, and Coping Humor Scale. The model was tested using structural equation modeling (SEM) and utilizing bootstrapping. The results of SEM showed that the effect of fear of COVID-19 on hopelessness was partly mediated by mindfulness and humor, and which was supported by bootstrapping. Therefore, higher fear of COVID-19 was associated with lower mindfulness and humor. In turn, lower mindfulness and humor were related with higher hopelessness. Findings are discussed in the context of COVID-19 and the hopelessness literature, and practical implications for counselors are also provided.

## Introducton

Since the spread of the novel coronavirus 2019 (COVID-19) became a pandemic, the attention of the world has rightly focused on protecting the population, minimizing the spread of the virus, and treating COVID-19 patients. During this period, governments around the world have implemented measures to limit contact between individuals, including imposition of quarantine, physical distancing, and social isolation. For example, in Turkey (where the present study was carried out), after the first confirmed case on March 10, 2020, some measures were implemented including online education in all schools, obligatory quarantine for individuals under 18 years and over 65 years, quarantine of 15 days in state accommodation units for individuals traveling from abroad, compulsory face mask use outside, travel restrictions within big cities, and flexible working practices for civil servants. While these measures are necessary to slow down the spread of COVID-19, the possible adverse mental health effects cannot be overlooked. Individuals who are in quarantine may experience boredom, anger, and loneliness. The symptoms of the viral infection together with the unfavorable effects of treatment may also result in worsening cognitive distress and anxiety among individuals (Xiang et al. 2020).

At the same time, worries and fears about stigmatization and discrimination because of COVID-19 can be a source of stress (Lin 2020). Additionally, the fear of COVID-19 causes individuals to be on alert to protect themselves and their loved ones which can lead to loneliness, fear, and panic in society (Yip and Chau 2020). Moreover, what is being discussed and disseminated throughout the media is also highly important and should be based on correct and reliable information during the COVID-19 crisis, since the public may rely on the media to get critical information to take necessary precautions. When the information is inaccurate or ineffectively communicated, it may lead to profound appraisals of threat which result in fear and psychological distress (Garfin et al. 2020).

As illustrated above, one of the most common psychological consequences of COVID-19 is fear. Extraordinary situations such as disease outbreaks and epidemics tend to trigger fear among individuals (Pakpour and Griffiths 2020). The unprecedented nature of the COVID-19 and the uncertainties about the future of the disease are likely to fuel the fear of COVID-19 (Asmundson and Taylor 2020). Mertens et al. (2020) reported that intolerance of uncertainty, health anxiety, risk for loved ones, and consulting increased information sources (e.g., regular media, social media, and professional media) are related to the fear of COVID-19. Their study also showed that individuals have concerns about the impact of the COVID-19 on the healthcare system, the economy, society, losing their job, and changes in daily routines, which can also fuel the fear of COVID-19. When individuals experience anxiety and uncertainty about their future, it is highly likely to lead to feelings of hopelessness for some individuals.

Hopelessness is composed of affective, cognitive, and motivational components, characterized by pessimistic cognitive schemas as well as negative expectations for the future (Beck et al. 1974). Hopelessness is the prominent feature of depressive symptoms (Young et al. 1992) and a high positive correlation has been reported between hopelessness, anxiety, and depression in the general population (Kocalevent et al. 2017).

Research has shown that hopelessness is a robust risk factor for suicide among clinical samples (Beck et al. 1985, 2006) and for suicidal ideation among non-clinical samples (Kliem et al. 2018). Hopelessness has been associated with early suicide attempts and suicidal thoughts in early adulthood (Troister et al. 2015). Similarly, among those who attempt suicide, high hopelessness has been associated with a high perception of stress (Elliott and Frude 2001). In relation to personality, it has been found that higher hopelessness is negatively associated with lower extraversion and lower conscientiousness and positively associated with higher neuroticism (Chioqueta and Stiles 2005; Velting 1999).

Suicidal ideation should be taken into account during an epidemic such as that of COVID-19. During the Ebola epidemic, it was reported that suicidal ideation increased among elderly individuals due to high anxiety (Yip et al. 2010). Suicide cases have also been reported during the COVID-19 pandemic (Bhuiyan et al. 2020; Goyal et al. 2020; Griffiths and Mamun 2020; Mamun and Griffiths 2020). In the course of a pandemic, one of the prominent reasons underlying suicidal ideation is likely to be hopelessness.

As would be expected, hopelessness is negatively related to positive psychological functioning-related concepts. For instance, hopelessness is negatively associated with life satisfaction (Kliem et al. 2018), is a negative predictor of quality of life among older adults (Scogin et al. 2016), and is a negative predictor of resilience towards stressful events (Hjemdal et al. 2012). In a study among military personnel, optimism was reported to be a key negative predictor of hopelessness (Bryan et al. 2013). Taking these findings into account, there is clearly a need for functional coping methods that can be used to mitigate hopelessness and stress during the COVID-19 pandemic. One such coping mechanism is humor. Consequently, in the present study, it was hypothesized that humor may mediate the relationship between fear of COVID-19 and hopelessness.

Humor is crucial in protecting the self and maintaining positive psychological functioning in daily life as well as coping with more traumatic experiences (Frankl 1963; Freud 1928). One of the possible reasons for this is that humor contributes to cognitive reappraisal during stressful situations (Kuiper et al. 1995). By interpreting a stressful condition as a positive challenge, using humor as a coping mechanism is an important self-protection strategy (Kuiper et al. 1993) because it had a stress-buffering effect (Fritz et al. 2017).

Furthermore, humor may be considered as an important identity protection mechanism. For instance, it has been reported that laughter is an important coping strategy for dealing with stressful situations such as racial micro-aggression (Houshmand et al. 2019). In a recent study conducted during the pandemic, humor was found to be associated with psychological well-being among individuals with a chronic illness and disability (Umucu and Lee 2020).

Given these findings, it can be assumed that humor can improve an individual’s behavior and thought repertoire and thus their coping skills in stressful conditions. Humor may facilitate alleviating the vicious circle of learned helplessness, which is the key vulnerability factor for depression (Abramson et al. 1978) and help eradicate hopelessness. Therefore, determining the buffering role of humor relation in the relationship between fear of COVID-19 and hopelessness is a worthy research area.

In addition to the use of humor, mindful awareness is also included in the proposed mediation model because it is an important stress reduction and self-regulation strategy (Brown and Ryan 2003; Brown et al. 2007; Kabat-Zinn 2003). Mindfulness is the purposeful directing of attention to the present experience (Brown and Ryan 2003) by inhibiting the rumination of past and future experiences (Blanke et al. 2019). It has been found that uncertainty during the COVID-19 pandemic can damage mental well-being by increasing rumination (Satici et al. 2020a). Researches have indicated that high mindful awareness is associated with high authenticity and meaning in life (Allan et al. 2015), as well as high hope and self-efficacy among school counselors (Ender et al. 2019). Another study found that mindfulness predicts resilience and needs satisfaction (Charbonneau 2019). In a study investigating the regulatory function of mindfulness, the mediating role of emotion and mood regulation in the relationship between mindfulness and depressive symptoms was reported (Jimenez et al. 2010). Considering all these findings, mindfulness is evidently an important source of psychological well-being, especially in non-Western cultures (Christopher 2018).

Mindfulness is an effective coping method used in overcoming traumatic processes. More specifically, it has been found to be an effective strengthening tool in the therapeutic process for trauma veterans (Lukoff and Strozzi-Heckler 2017), and an effective defense method against mortality salience (Niemiec et al. 2010). The COVID-19 pandemic has had a traumatic effect on individuals globally, and therefore, it is predicted that mindfulness may be an effective method of overcoming hopelessness-related cognitions in the current climate.

### The Present Study

In Turkey (at the time of writing, October 8, 2020), there had been over 6800 COVID-19 deaths and over 284,000 confirmed cases (Ministy of Health 2020). In terms of total cases, Turkey is ranked eighteenth among all countries (Worldometer 2020). With the rate of spread of infection currently increasing again at the time of writing, measures such as the obligation to wear masks in all areas outside of the home have been re-applied. During the COVID-19 pandemic, epidemic deaths, countrywide isolation, and wide-scale job losses have emerged as crucial risk factors in poor psychological well-being (Lee et al. 2020). In their studies, Lee et al. (2020) found a strong positive relationship between COVID-19 anxiety and hopelessness as well as suicidal ideation. Therefore, in order to control the psychological effects occurring as a result of the pandemic, there is an urgent need to combat hopelessness-related cognitions. Based on this premise, the present study’s aim was to examine the relationships among fear of COVID-19, mindfulness, humor, and hopelessness in a Turkish cross-sectional sample. Therefore, investigating the mediating roles of mindfulness and humor (as coping strategies) in the relationship between fear of COVID-19 and hopelessness was considered worthy of empirical research. Consequently, the following hypotheses (*H*s) were proposed:

1. Fear of COVID-19 will be positively associated with hopelessness.
2. The association between fear of COVID-19 and hopelessness will be mediated by mindfulness and humor.

## Method

### Participants and Procedure

Online convenience sampling was utilized to recruit participants for the study. The participants comprised 786 individuals from 71 of 81 cities in Turkey (562 females [71.5%] and 224 males [28.4%]). Their ages ranged from 18 to 67 years, with a mean of 24.02 years (SD = 7.82). In terms of socio-economic status, 99 were low status (11.7%), 658 were medium status (83.8%), and 36 were high status (4.5%). More than half of the participants had completed undergraduate education (*n* = 499, 63.5%). The highest education level of the remaining participants was 22 primary school (2.8%), 33 secondary schools (4.2%), 124 high school (15.8%), 104 associate degree (13.2%), and four postgraduate (0.5%).

The data were collected in May 2020 utilizing an online survey, and the research procedure involved different steps. The study was approved by the Artvin Coruh University Scientific Research and Ethical Review Board (REF: 78646441-050.01.04-E. 5373), and the work was conducted in accordance with the Declaration of Helsinki. The survey was advertised to the participants via the social media accounts used by the research team. Participants were told that scientific research was being conducted examining the psychological impact of COVID-19. The researchers advertised the survey on their social media accounts and asked for help in both volunteer participation and distribution. During data collection, emphasis was placed on voluntary participation. Also, the data were collected anonymously, and the participants were told that they could withdraw from the study at any time. Informed consent was provided by all participants. There were no missing data since all the questions on all surveys were complete.

### Measures

*Fear of COVID-19* was assessed using the Fear of COVID-19 Scale (FCVS-19; Ahorsu et al. 2020). The scale contains seven items (e.g., “My hands become clammy when I think about coronavirus 2019”) which were answered using a 5-point Likert-type scale ranging from 1 (“strongly disagree”) to 5 (“strongly agree”). The Turkish version was used in the present study (Satici et al. 2020). The Turkish version of the FCVS-19 in the adaptation study had a good fit to the data (*χ*^2^_(13, *N* = 1304)_ = 299.47, *p* < .05; GFI = .936; SRMR = .061; NFI = .912; CFI = .915). Scores range from 7 to 35 with higher scores indicating higher fear of COVID-19. In the present study, the Cronbach’s alpha coefficient for the FCVS-19 was very good (0.88).

*Hopelessness* was assessed using the Beck Hopelessness Scale (BHS; Beck et al. 1974). The scale contains 20 items (e.g., “My future seems dark to me”) which are answered in a true/false format. The Turkish version was used in the present study (Durak 1993). During the development of the BHS into Turkish, Durak (1993) did not carry out any confirmatory factor analysis. Therefore, CFA was conducted within the scope of the present study, and there was a good fit to the data (*χ*^2^_(170, *N* = 786)_ = 509.10, *p* < .05; GFI = .935; SRMR = .061; AGFI = .920). Scores range from 0 to 20 with higher scores indicating higher hopelessness. In the present study, the Cronbach’s alpha coefficient for the BHS was very good (0.86).

*Mindfulness* was assessed using the Mindful Attention Awareness Scale (MAAS; Brown and Ryan 2003). The scale contains 15 items (e.g., “I could be experiencing some emotion and not be conscious of it until some time later”) which are answered on a 6-point Likert-type scale ranging from 1 (“almost always”) to 5 (“almost never”). The Turkish version was used in the present study (Ozyeşil et al. 2011). The Turkish version of the MAAS in the adaptation study had a good fit to the data (*χ*^2^_(90, *N* = 278)_ = 187.811, *p* < .05; GFI = .93; RMSEA = .06; AGFI = .91). Scores range from 15 to 75 with higher scores indicating greater mindful awareness. In the present study, the Cronbach’s alpha coefficient for the MAAS was very good (0.80).

*Humor* was assessed using the Coping Humor Scale (CHS; Martin and Lefcourt 1983). The scale contains seven items (e.g., “I often lose my sense of humor when I am having problems”) which are answered on a 4-point Likert-type scale ranging from 1 (“strongly disagree”) to 4 (“strongly agree”). The Turkish version was used in the present study (Yerlikaya 2009). During the development of the CHS into Turkish, Yerlikaya (2009) did not carry out any confirmatory factor analysis. Therefore, CFA was conducted in the present study, and there was a good fit to the data (*χ*^2^_(14, *N* = 786)_ = 138.90, *p* < .05; GFI = .954; SRMR = .064; AGFI = .908). Scores range from 7 to 28 with higher scores indicating greater humor coping. In the present study, the Cronbach’s alpha coefficient for the CHS was good (0.71).

### Data Analysis

In the present study, structural equation modeling (SEM) was used. SEM is one of the most popular quantitative methods in social sciences (Kaplan 2001). SEM “is a multivariate, hypothesis-driven technique that is based on a structural model representing a hypothesis about the causal relations among several variables” (Stephan and Friston 2009, p. 393). In this study, fear of COVID-19, hopelessness, mindfulness, and humor were regarded as latent variables. Therefore, a two-step SEM procedure suggested by Anderson and Gerbing (1988) was carried out to analyze the mediation roles. First, the measurement model was examined. Then, the structural model was tested using maximum likelihood estimation in the AMOS Graphics software. For evaluation of the goodness-of-fits model in SEM, standard criteria were utilized: comparative fit index (CFI), Tucker-Lewis index (TLI), and goodness of fit (GFI) above .90; standardized root mean square residual (SRMR) and root mean square error of approximation (RMSEA) below .08 indicate acceptable fit (Hu and Bentler 1999). Utilizing recent recommendations (Hayes 2018), the mediating role of fear of COVID-19 was investigated by examining the 95% upper and lower limits of bootstrapping confidence intervals (CIs) of the indirect effects. Bootstrapping is a versatile method that has become frequently used in contemporary research. This method is particularly useful in investigating complex and highly context-dependent properties (Hayes 2018).

## Results

### Correlations

Correlations were computed to investigate the bivariate relationships between fear of COVID-19, hopelessness, mindfulness, and humor (see Table 1). Fear of COVID-19 was positively associated with hopelessness (*r* = .27, *p* < .001) and negatively associated with mindfulness (*r* = − .25, *p* < .001) and humor (*r* = − .23, *p* < .001). Hopelessness was negatively associated with mindfulness (*r* = − .35, *p* < .001) and humor (*r* = − .35, *p* < .001).

**Table 1:** Descriptive statistics, reliabilities, composite reliability (CR), average variance extracted (AVE), and discriminant validity

Variable	M	SD	Skewness	Kurtosis	*α*	*ω*	CR	AVE	1	2	3	4
1. Fear of COVID-19	17.76	6.01	.21	− .47	.88	.88	.87	.76	(.87)			
2. Hopelessness	6.62	4.63	.81	− .29	.86	.87	.85	.66	.27**	(.81)		
3. Mindfulness	55.28	10.79	.11	.09	.80	.80	.79	.56	− .25**	− .35**	(.75)	
4. Humor coping	18.49	2.92	.05	.42	.71	.72	72	.55	− .23**	− .35**	.22**	(.74)

Diagonals (in parenthesis) represent the square root of AVE while off diagonals represent correlations

### Structural Equation Modeling

#### Measurement Model

Four latent constructs (fear of COVID-19, hopelessness, mindfulness, and humor coping) and ten observed variables were included in the measurement model. The fit statistics indicated that the measurement model provided a good fit to the data. The ratio of the *χ*^2^ to the degrees of freedom (*χ*^2^/df = 2.64) and the goodness-of-fit indices (CFI = .98, TLI = .97, GFI = .98, SRMR = .031, RMSEA = .046) were within the cutoff ranges recommended by Hu and Bentler (1999). All factor loadings were significant and varied between .74 and .96. The reliability coefficients were above .70 (varying from 0.71 to 0.81) and were therefore at an acceptable level. The measurement model was also assessed to check the validity of items representing each latent construct (Fornell and Larcker 1981). For each latent variable, composite reliability was greater than 0.70 (varying from 0.72 to 0.87), and the average variance extracted exceeded 0.50 (varying from 0.55 to 0.76), indicating that each construct possessed high internal consistency (see Table 1).

#### Structural Model

**Fig. 1:**
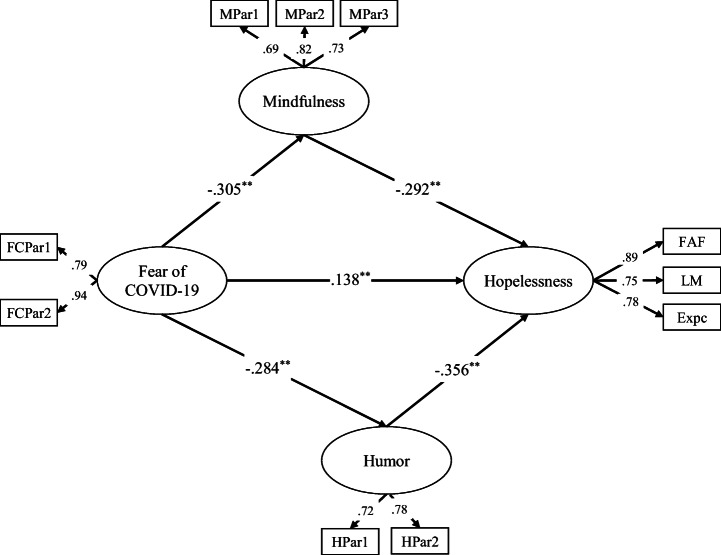
Standardized factor loading for the partially mediated structural model. *N =* 786; **p < .001. FCPar, parcels of fear of COVID-19; MPar, parcels of mindfulness; HPar, parcels of humor; FAF, feeling about the future; LM, loss of motivation; Expc, expectation

During the testing of the structural models, gender was added to the model as a control variable. In the first step, the direct effect of the predictor (fear of COVID-19) on the dependent variable (hopelessness) in the absence of mediators (mindfulness and humor) was tested. The overall fit of the fully mediated model to the data was acceptable (*χ*^2^/df = 4.07, CFI = .96, TLI = .94, GFI = .96, SRMR = .063, RMSEA = .063, AIC = 214.66, ECVI = .273). After that, a partially mediated model that contained mediators (mindfulness and humor) and a direct path from fear of COVID-19 to hopelessness was tested. The partially mediated model showed a very good fit to the data (*χ*^2^/df = 3.87, CFI = .96, TLI = .95, GFI = .97, SRMR = .061, RMSEA = .061, AIC = 205.12, ECVI = .261). Therefore, it was seen that the fit indexes of both partial and full mediation models were acceptable. The chi-square difference test and AIC-ECVI values were examined for which model to choose. Based on the chi-square difference test, the direct path made a significant contribution to the model (Δ*χ*^2^ = 11.54, df = 1, *p* < .001). Also, the partial model’s AIC and ECVI were lower than the full model’s AIC and ECVI. Consequently, partial models were preferred (see Fig. 1). In addition, a statistical power analysis was performed for the effect size. This was 1.0, which is considered extremely large using Cohen’s (1988) criteria.

#### Bootstrapping

The partially mediated model was tested for significance using the bootstrap estimation procedure (a bootstrap sample of 10,000 was specified). Direct, indirect, and total effects were given in Table 2. Fear of COVID-19 had a significant direct effect on hopelessness (*β* = .079; 95% CI = .043, .119), mindfulness (*β* = − .398; 95% CI = − .496, − .303), and humor (*β* = − .140; 95% CI = − .192, − .086). Hopelessness was directly affected by mindfulness (*β* = − .128; 95% CI = − .160, − .096) and humor (*β* = − .128; 95% CI = − .525, − .292). Additionally, the indirect effect of fear of COVID-19 on hopelessness via mindfulness was 0.051 (95% CI = .036, .068). The indirect effect of fear of COVID-19 on hopelessness via humor was 0.058 (95% CI = .040, .081). Therefore, higher fear of COVID-19 was also associated with lower mindfulness and humor and, in turn, lower mindfulness and humor were related with higher hopelessness.

**Table 2:** Direct, indirect, and total effects for the final model

Model pathways	Estimated	95% CI	
		Lower	Upper
Direct effect			
Fear of COVID-19 ➔ hopelessness	.079	.043	.119
Fear of COVID-19 ➔ mindfulness	− .398	− .496	− .303
Fear of COVID-19 ➔ humor	− .140	− .192	− .086
Mindfulness ➔ hopelessness	− .128	− .160	− .096
Humor ➔ hopelessness	− .128	− .525	− .292
Indirect effect			
Fear of COVID-19 ➔ mindfulness ➔ hopelessness	.051	.036	.068
Fear of COVID-19 ➔ humor ➔ hopelessness	.058	.040	.081
Total effect	.187	.149	.224

## Discussion

Two hypotheses were formulated to test direct and indirect relationships and to test the mediation model proposed within the scope of the study. The results showed that fear of COVID-19 was directly associated with hopelessness. At the same time, COVID-19 fear predicted hopelessness over humor and mindfulness. Therefore, the proposed mediation model was partially accepted.

The hypothesis that concerned the relationship between fear of COVID-19 and hopelessness (H_1_) was confirmed. This finding is in line with the research findings showing the relationship between COVID-19 anxiety and hopelessness (Lee et al. 2020). Moreover, there is a high negative correlation between hopelessness and anxiety more generally (Kocalevent et al. 2017). The findings here overlap with studies showing that suicidal ideation, which is closely related to hopelessness, may increase during an epidemic (Bhuiyan et al. 2020; Goyal et al. 2020; Griffiths and Mamun 2020; Yip et al. 2010). In addition, deterioration in financial conditions and job loss have been found to be the main risk factors in prolonging hopelessness (Haatainen et al. 2003). Consequently, global financial uncertainty is likely reinforcing the fear of COVID-19 and hopelessness. In addition, although the media is a source of information, the frequent presence of COVID-19-related information in the media may have a paradoxically negative effect (Garfin et al. 2020). Frequent media coverage of global COVID-19 news and associated problems such as economic uncertainly and deaths may activate hopelessness-related cognitive schemas.

Within the scope of the research, H_2_ which proposed the mediation of humor in fear of COVID-19 and hopelessness was partially confirmed. This finding showed the buffering role of humor (which has been a timeless coping method among humans) between fear of COVID-19 and hopelessness. Accordingly, this finding of the study concurs with the findings of research showing the role of humor in coping with stress (Bizi et al. 1988; Ferguson 2016; Fritz et al. 2017). From an evolutionary perspective, humor is an important tool in facilitating survival (Caron 2002). Therefore, as a result of the present study’s findings, it can be suggested that humor is a resilience factor in the context of COVID-19 fear and hopelessness during the pandemic. Additionally, humor, which is an important determinant for cognitive re-appraisals (Kuiper et al. 1995), predicted hopelessness in the present study. Similarly, it may be speculated that humor could change pessimistic explanatory styles by using cognitive processes more effectively.

H_2_ examined the mediation of mindful awareness in the relationship between fear of COVID-19 and hopelessness and was also partially accepted. This finding is consistent with research findings showing the function of mindfulness in affect regulation (Jimenez et al. 2010) and as a predictor of mindfulness on hope (Ender et al. 2019). Therefore, mindful awareness may play an important role in regulating the fear of COVID-19 and in reducing hopelessness. In addition, considering findings showing that an important function of mindful awareness is decreasing rumination (Blanke et al. 2019) and rumination is an important predictor of COVID-19 fear and well-being during the pandemic (Satici et al. 2020a), it may be that (like rumination) mindfulness is also a protective strategy for hopelessness.

### Limitations

There are a number of limitations to consider when interpreting the findings. Firstly, the study was carried out utilizing a non-clinical sample. The research needs to be retested among clinical participants because they more likely to experience chronic fear and despair during the pandemic. Secondly, it is not possible to draw causal conclusions from the research results given the cross-sectional nature of the study. İn order to gain insight on more causal inferences among the variables, longitudinal and experimental studies are needed. Consequently, humor and mindfulness priming studies should be carried out. The study also used a convenience sample comprising self-report data and as such there are well-known common method biases that may have influenced the finding. Finally, groups exposed to potential macroaggression such as ageism, ableism, classism, and racism, there may be an intersection of already existing stressors alongside COVID-19-related stress. Therefore, in future studies, variables that may be effective in dealing with hopelessness in the COVID-19 process may be examined in these specific groups instead of the general population. Finally, because of the nature of convenience sampling, equality in gender distribution was not achieved. However, the present study included gender as a control variable in the structural model. In order to overcome this limitation, gender distribution in the context of nationally representative study should be attempted in future studies. Additionally, this limitation could be compensated for by utilizing meta-analysis in future studies.

## Conclusion

The present study showed that COVID-19-related fear is a powerful positive predictor of hopelessness. This predictive relationship is partially buffered by mindfulness and humor. In line with the findings of the research, mental health professionals should utilize online bibliotherapy practice comprising humorous literature during the pandemic. Similarly, group-based cine therapy applications may be prepared online by making use of various humorous films and television. Moreover, a set of culturally responsive mindfulness activities could be established which might be followed through the use of smartphone applications. Moreover, the research here indicates that humor and mindfulness (which are both relatively alterable as well as being positive concepts) could be utilized by health promotion practitioners at a general level and by therapists at an individual level to reduce the negative psychological effects of the COVID-19 pandemic. Additionally, the effect of techniques used in existential anxiety and stress interventions in third-generation cognitive behavioral therapies could also be specifically utilized among those with severe psychological consequences involving the fear of COVID-19.
